# Intervention-Based Stochastic Disease Eradication

**DOI:** 10.1371/journal.pone.0070211

**Published:** 2013-08-05

**Authors:** Lora Billings, Luis Mier-y-Teran-Romero, Brandon Lindley, Ira B. Schwartz

**Affiliations:** 1 Department of Mathematical Sciences, Montclair State University, Montclair, New Jerey, United States of America; 2 U. S. Naval Research Laboratory, Nonlinear System Dynamics Section, Plasma Physics Division, Washington, DC, United States of America; 3 Johns Hopkins Bloomberg School of Public Health, Department of International Health, Baltimore, Maryland, United States of America; University of Leeds, United Kingdom

## Abstract

Disease control is of paramount importance in public health, with infectious disease extinction as the ultimate goal. Although diseases may go extinct due to random loss of effective contacts where the infection is transmitted to new susceptible individuals, the time to extinction in the absence of control may be prohibitively long. Intervention controls are typically defined on a deterministic schedule. In reality, however, such policies are administered as a random process, while still possessing a mean period. Here, we consider the effect of randomly distributed intervention as disease control on large finite populations. We show explicitly how intervention control, based on mean period and treatment fraction, modulates the average extinction times as a function of population size and rate of infection spread. In particular, our results show an exponential improvement in extinction times even though the controls are implemented using a random Poisson distribution. Finally, we discover those parameter regimes where random treatment yields an exponential improvement in extinction times over the application of strictly periodic intervention. The implication of our results is discussed in light of the availability of limited resources for control.

## Introduction

Understanding the processes underlying disease extinction is an important problem in epidemic prediction and control. Currently, total eradication of infectious disease is quite rare, but continues to be a major theme in public health. Temporary eradication, sometimes called fade out, tends to happen in local spatial regions, and may be followed by the reintroduction of the disease from other regions [1], [2], [3]. In the case of diseases that possess co-circulating strains such as influenza [4], or dengue fever which has up to four strains [5], extinction may occur in one or more strains while the others persist. Infectious disease transmission is also conjectured to be responsible for certain species extinction [6], [7]. Recently, large scale amphibian species have had major declines in population, which have been linked with the spread of disease [8].

One main reason that diseases go extinct is due to the stochasticity that is inherent to populations of finite size [9]. As a disease evolves in a large finite population, there is the possibility of insufficient transmission for it to stay endemic. Therefore, in finite time, the number of infectious individuals can go to zero and the disease dies out [10], [11], [12]. Other mechanisms that enhance extinction include small populations and resource competition [13], as well as heterogeneity in host–vector models [14]. Extinction or fade out may also occur within host, as in the theoretical study of spontaneous virus clearance of Hepatitis C and HIV [15].

To properly model the random interactions occurring in populations, the study of disease extinction requires a stochastic modeling approach. There are numerous studies from time series analysis and epidemic modeling supporting stochastic fluctuations due to random interactions [16], [17], [18], [19]. The fluctuations may act as an effective force that drives the disease to vanish [20]. While each stochastic realization is Markovian, extinction arises from an organized set of fluctuations which may overcome the instability of the extinct state. The goal is to identify the set of fluctuations which result in the pattern of the noise necessary to drive the system out of equilibrium from the attracting state to the extinct state. The optimal path depends on boundary conditions in the asymptotic limit of past and future history, and represents the most probable trajectory from the endemic to the extinct state.

We remark here that although escape has been considered for systems of Langevin type, the theory we present in this paper is for discrete finite populations modeled as a master equation. In continuous systems, a rigorous theory of escape rates for systems driven by white Gaussian noise was developed by Freidlin and Wentzell [21]. It was also found that the escape rates should display a number of universal features, including scaling behavior near bifurcation points [22], [23], which has been confirmed by many experiments [24], [25], [26], [27]. Conversely, Allen and Burgin [10] used Markov chain analysis to approximate the duration of an epidemic in discrete time models. More recently, it was shown that the state of the system is coupled to a deterministic model of the noise shape [28]. In this setting, the optimal path is an unstable object, but may be associated with the dynamical systems idea of having maximum sensitive dependence to initial conditions [29].

Treatment programs are common methods used to speed up the extinction of a disease in a population [30]. In this paper, we aim to quantify how random treatment programs increase average extinction rates. We focus on a class of diseases with no immune response. Models with no immunity are suitable for many bacterial infections, such as meningitis, plague, and venereal diseases, as well as certain protozoan illnesses, such as malaria and sleeping sickness [31].

In general, little work has been done in analyzing stochastic models with random treatment intervention. In this context, we assume that treatments would be applied to infected individuals, removing them from that group. Most intervention schedules are designed as periodic, especially for childhood and seasonal diseases [32]. Each intervention typically has a prescribed (deterministic) schedule, or distribution, of treatment doses, but the extinction event is still random. Similarly, there has been work on using vaccination distributions as a control mechanism [33] and recently, this idea has been extended to stochastic models in [34], [35].

Thus, one of the main problems in understanding treatment scheduling is that deterministic schedule models are *not* an accurate representation of the process. A more realistic scenario is that, on average, treatment scheduling has a mean period or cycle, but is itself a random process. In this paper, we study a randomly distributed treatment program of infected individuals. We are interested in evaluating treatment distributions by minimizing the mean time to extinction for the disease. Running simulations are computationally expensive and sensitive to population size. The theory presented in this paper provides an alternate method to approximate the mean time to extinction. In our models, we identify conditions for which the escape rate theory applies and control strategies are effective. In particular, we derive explicit scaling functions of the exponent of the mean time to extinction in terms of basic reproductive number and mean treatment levels. We also identify the most effective treatment schedules. Then, we compare the theory against numerical simulations for verification.

## Methods

In this paper, we use the stochastic SIS compartmental model as a basic example to clearly demonstrate our mathematical methods analytically and numerically. The methods can be extended for use in more complex models, as necessary for a disease of interest. The SIS model tracks the number of individuals in a population of size 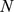 in one of two states: susceptible (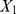) or infected (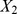). In this model, we assume that the individuals become susceptible to the disease again upon recovery. The number of individuals in each state changes as birth, death, infection and recovery events occur. They are quantified by the following transition rates. If a susceptible comes in contact with an infected individual, the healthy individual may become infected. We use a mass action term with the contact rate 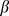 to describe the flow of newly infected individuals from the susceptible group. We assume infected individuals recover at rate 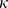 and immediately re-enter the susceptible group. New susceptible individuals are born at a rate 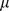, and both susceptible and infected individuals have a death rate of 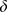. In this model, we assume that the individuals recover from the disease without significant mortality. We also assume that the population is constant over time, on average, and therefore set the birth rate is equal to the death rate, so 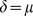. This assumption allows steady states in the model, for which we can analyze the stability.

Associated with the parameters for a particular disease is the basic reproduction number, 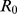, which defines on average how many new cases appear over one infectious period per infective [1]. Deterministically, when 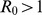, the disease persists. In other words, the extinct state is unstable and the number of infectious individuals approaches a limit called the endemic state. The 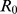 for a particular disease can be approximated from data. For example, it was approximated from the epidemiological data from England and Wales that the serogroup C meningococcal disease had 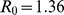, [36]. In Africa, some malaria 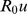 estimates are close to one, but others can be as high as 3,000 [37]. This variation is attributed to environmental temperature variations and mosquito biology [38]. Therefore, several groups have identified the applicability for methods to analyze extinction in finite populations near the bifurcation point 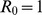. (See the review in [39].) In both basic SIS [40], [41] and SIR [42] models, the mean times to extinction were analyzed as a function of 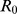 very close to one. The range of parameters in both papers is assumed to model extremely slow disease propagation in large population limits. In this paper, we continue with the analysis for a non-specific disease with 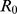 close to one, noting that parameters can be adjusted for a disease of interest.

For disease control, the stochastic model assumes a treatment schedule that occurs at randomly chosen times with a frequency 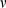 times per year. Each time the treatment is applied, a fraction 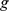 of all infected individuals recover and flow back into the susceptible class. This assumes the treatment has 100% efficacy. To study the effect of treatments that are not as effective, a prefactor for 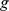 could be added to capture the smaller efficacy. That case is similar to studying a smaller value for 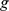, which is included in the parameter range 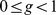 and therefore we do not study this issue separately.

We use the master equation approach to describe the time evolution of the stochastic system. The general theory of applying the WKB method to finite populations begins by assuming that the population of 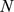 individuals described by a state vector 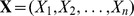. Let the random state transitions governing the dynamics be described by the transition rates 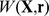, with 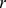 representing the increment in the change of each component of 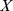. Also, let the probability of finding the system in state 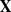 at time 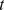 be 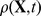. We assume that the system possesses a single, strictly stationary solution for the probability density, 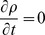, that corresponds to the extinct state, where one or more of the 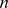 components of the state vector 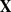 are equal to zero. The stability of this solution is essential, since convergence to this stationary solution represents the set of possible trajectories that lead to extinction.

When the probability current at the extinct state is sufficiently small, there will exist a quasi-stationary probability distribution with a non-zero number of infected individuals that decays into the stationary solution over exponentially long times. The rate at which the extinction of infected individuals occurs may be calculated from the tail of the quasi-stationary distribution. It has been shown that a WKB approximation to the quasi-stationary distribution allows one to approximate the mean-time to extinction with high accuracy for a sufficiently large population [43], [40], [41].

Approximating the probability by 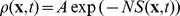 for the normalized state 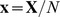 (e.g., in an epidemic model, the fraction of the population in the various compartments), we form the Hamilton-Jacobi equation: 
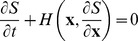
. In analogy to Hamiltonian mechanics, the functions 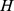 and 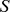 are called the Hamiltonian and the action, respectively. Let 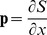, which is called the momentum conjugate to 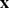. Using the scaled transition rates 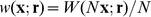, the Hamiltonian function is 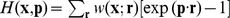, and we analyze the system using the characteristic equations: 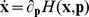, 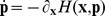. For a more detailed description of the WKB method and other applications, see [44], [45], [40].

## Model 1: Constrained SIS model with treatment

In the first model, we approximate the SIS dynamics by reducing the dimension of the problem. Assume the average population size is 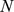 and constrain the population size such that 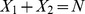. Therefore, we can consider the dynamics of the constrained SIS model in terms of infected individuals, 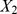. We need only to consider the following transition rates, which describe how individuals enter and leave the infected state:



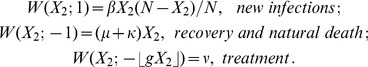
.

Since the population variable in the master equation is integer-valued, we choose to keep the integer part 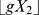 of 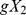 (rounding down). Using these transition rates, the master equation for the constrained SIS stochastic process is
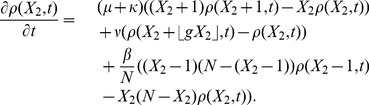
(1)


Note that for any particular realization of the master equation, the treatment ceases to have an effect whenever 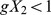 (i.e., 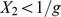) since for all those numbers of infecteds 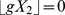. It is well known that the basic reproduction number for the deterministic SIS model without treatment (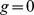) is 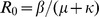. With treatment, we define the reproduction number 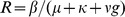.

Equation (1) will always possess as a solution a stationary distribution with the probability of observing zero infected individuals 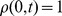, which we identify as the extinct state 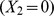. If 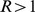 and 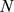 is large enough, Eq. (1) will also possess a quasi-stationary solution with an infected fraction fluctuating around an endemic state. Hence, if 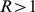 the disease can spread through a population and is considered endemic.

Next, we rescale the state variable by the population by using the normalized variable 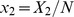. Therefore, the Hamiltonian function for the SIS model is
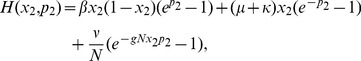
(2)and the associated Hamiltonian system is



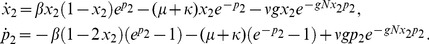
(3)The Hamiltionian system has three steady states of interest. Associated with deterministic dynamics (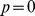) are the disease free equilibrium is 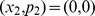 and the endemic state is 
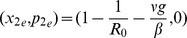
. In addition, there is a stochastic die out state, 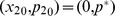, with 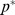 implicitly defined by
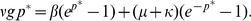
(4)


While stochastic die out state is similar to the disease free equilibrium having 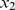 = 0, the difference is that momentum is nonzero. In an extinction event, the solution starts near the endemic state and approaches the stochastic die out state, not the disease free equilibrium.

Note that the endemic state exists only if 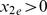. In addition, the endemic state has zero momentum, which is consistent with our expectation that the probability distribution have a maximum at 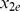 and hence 
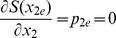
. Since the variables 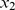 and 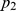 of the WKB approximation are not restricted to integer values, here the rounding of 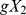 poses no problem. However, this means that in the WKB framework, the treatment pulses have an effect at arbitrarily low values of 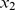, in contrast to the master equation framework where, because of the rounding, treatment stops being applied whenever 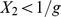.

### Model 2: Full SIS Model with Treatment

The second model is the unconstrained SIS treatment model in two-dimensions. We calculate 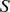 and 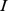 separately and allow the population fluctuation about 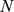. If the fluctuations are small compared to 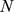, the system will behave like the one-dimensional approximation.

For the two-dimensional model, let the state vector be 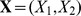 and the transition vector be 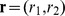. The changes in the susceptible and infected populations for a single transition are represented by the transition rates:



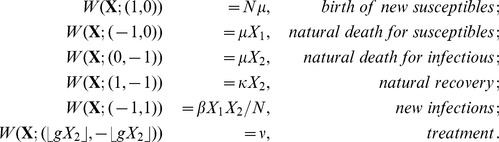
.

Here, as in Model 1, the non-integer quantity 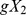 is rounded down to 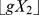. Again, rescale the state variable by the population and use the normalized vector 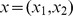, with 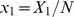 and 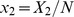. Using the definition of the master equation, the Hamiltonian in normalized variables is
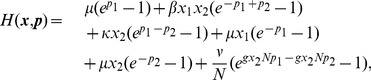
(5)and the associated Hamiltonian system is



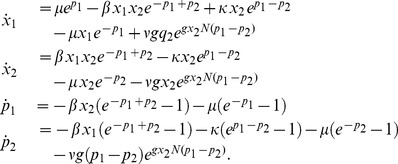
(6)Note once more that in the WKB framework, the treatment pulses have an effect for arbitrarily small 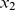. For this Hamiltonian system, the endemic state is located at the point
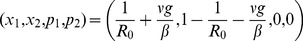
(7)and the stochastic die out state is 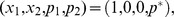 with 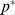 defined implicitly as in Eq. (4).

## Results

We now use these Hamiltonian models to approximate the mean time to extinction. Topologically, the solution that describes an extinction event in the Hamiltonian system will connect the endemic state (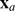) and stochastic die out state (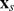). The connecting manifold is, in fact, the most probable path to extinction when the stochastic system starts initially at the endemic state [40], [41]. This set of points is called the optimal path. Points on the path will also satisfy the Hamiltonian on the energy surface 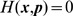 since it is a solution to the time-independent version of Hamilton-Jacobi equation.

From the definition of the momentum, 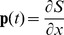, the action along the optimal path can be approximated by
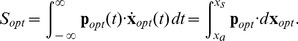
(8)


Using this quantity, we approximate the mean time to extinction by evaluating
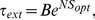
(9)where 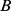 is a prefactor that depends non-exponentially on the system parameters and on the population size. An accurate approximation of the mean-time to extinction depends on obtaining 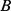 [46].

It is usually not a trivial task to identify the set of points that describe the optimal path. In some cases, it can be found analytically. One example is Model 1 with 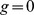, since Eq. (2) has an explicit solution for 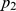 when constrained to 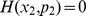. An alternative approach is approximating the solution asymptotically. There are also several numerical approaches. One common method is to treat the system as a two point boundary value problem and solving using a shooting method [47]. In this paper, we use a generalized Newton’s method that involves iterating an initial guess of the solution in the entire time domain [48]. Our initial guess must satisfy the property that the solution will stay asymptotically near the steady states except for a small, continuous, transition region between the two. This iterative procedure requires discretizing the model differential equations in time, using a second order approximation for the derivatives, and then solving the entire resulting system of nonlinear algebraic equations simultaneously.

Equation (9) holds if and only if a quasi-stationary distribution exists. This is the case if the time to extinction is exponentially long, i.e., 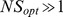. Assuming that an endemic state does exist (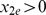); 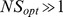 will be satisfied for 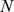 sufficiently large or, for fixed 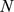, for an 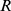 sufficiently large and 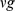 sufficiently small. The last conditions on the parameters mean that the disease should be highly transmissible and that the treatment should not be too intense. See Text S1, for a more detailed treatment on the necessary conditions for the quasi-stationary solution to exist.

### Model 1

Because the Hamiltonian system for the constrained model is in two dimensions, the first approximation to the action path simplifies to
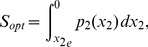
(10)with 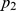 explicitly as a function of 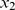, evaluating the integral along the optimal path. The Hamiltonian function of Eq. (2) does not allow for an algebraic solution for 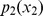 from the equation 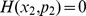 that describes the path connecting the endemic state to the extinct state when 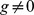. Therefore, the integral in Eq. (10) must be approximated.

For this model, an asymptotic approach can be used to approximate the action along the optimal path to extinction. We assume 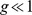, which implies small treatment pulses. We expand 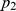 in 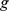 and substitute this expression into the equation 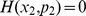. The resulting expansion is
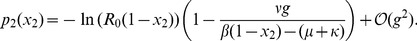
(11)


**Figure 1:**
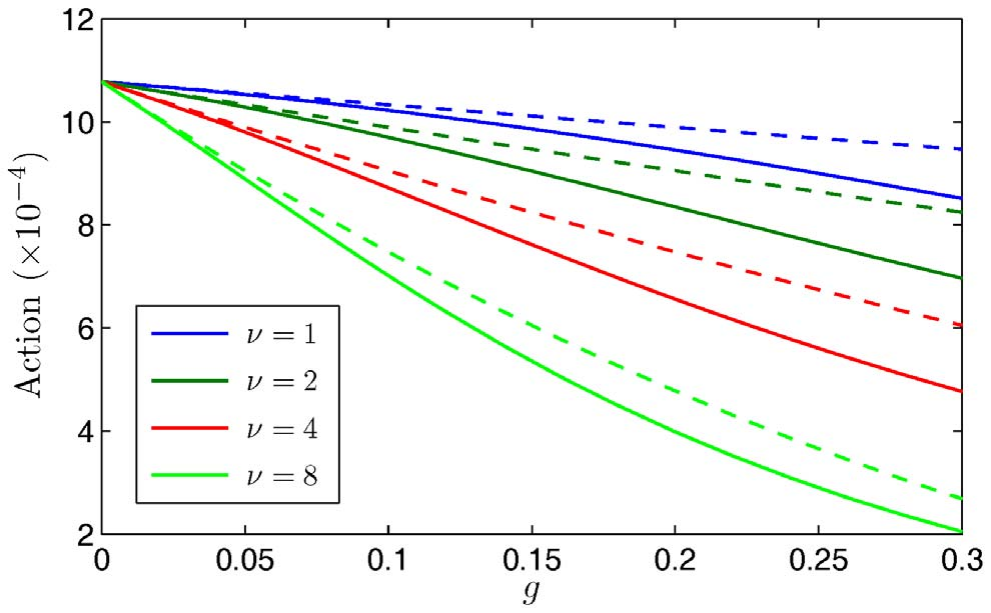
Comparing quantitative approximations of the action. For Model 1, plot of the numerical approximation of the action (dashed curve) and the asymptotic approximation (solid curve) as a function of the treatment, 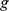. In this example, we use the parameters 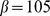 year^−1^ and 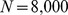 people. As expected, the best agreement is for small 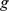.

The first term in the expansion 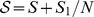 is given by Eq. (10). In [49], the second term in the expansion of the action in powers of 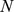 is given as a more complicated integral along the path. We expand the two integrals giving 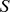 and 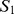 in powers of 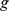 and evaluate them in closed form using computer algebra software. If we compare this asymptotic approximation to the numerical approximation for the action along the optimal path and evaluate Eq. (9), we see excellent agreement as shown in the example in Figure 1. In this example, we set the birth rate 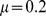 year^−1^ and recovery rate 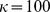 year^−1^. These generic parameters are chosen to represent a slowly spreading disease, but with a large 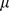 to demonstrate the scalings. For the remainder of the paper, we will use these parameters in examples for both models. These values provide results that can be clearly visualized and easily reproduced.

Note that while the action does not depend on the size of the population, to first order, the mean time to extinction does. The population size must be large enough for the system to be quasi-stationary. Our model assumes that disease extinction is a rare event, which occurs in the tail of the distribution described by 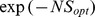. Conversely, the peak of the distribution occurs at the endemic state. As 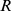 decreases to one, the distance between the endemic state and the disease free equilibrium decreases and the probability of the system having zero individuals in the infected state becomes significant. Therefore, the exponent must be large and negative, or equivalently the action must be sufficiently large compared to the population so that 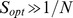.

**Figure 2:**
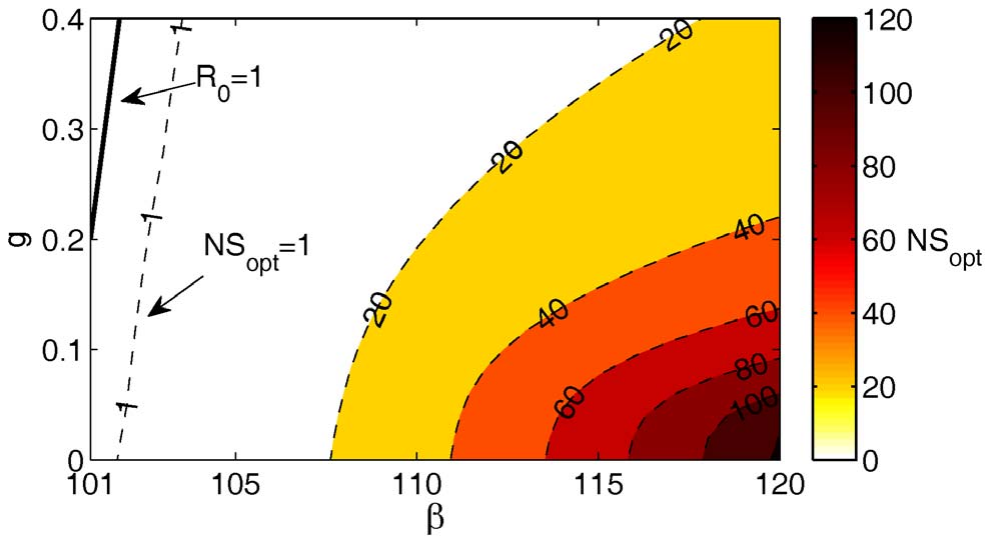
Checking the threshold for quasi-stationarity. A contour plot of 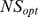 for Model 1 as we vary treatment, 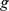, and the contact rate, 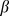. The darker colors represent larger values of 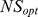. In this case, the treatment frequency is 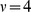 year^−1^ and 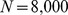 people. The solid black curve denotes where 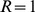, the boundary for the existence of the endemic state in the mean-field model. Quasi-stationarity holds for 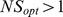. Therefore, between 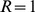 and 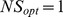, a larger population would be necessary for the mean-field equations and the stochastic model to agree.

To quantify where the system is quasi-stationary, we evaluate evaluate 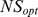 using the numerical approximation of the optimal path and Eq. (10). In Figure 2, we show a contour graph of 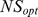 with frequency 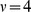 year^−1^ and a population of 8,000. The parameters region to the right of 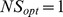 will allow quasi-stationarity, meaning extinction will lie in the tail of the distribution. Between 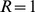 and 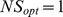, a larger population would be necessary for the mean-field equations and the stochastic model to agree. As the population increases (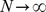), the 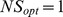 boundary will move to the left, towards the 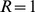 boundary. For 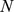 large enough, one expects these two curves to be close to each other and nearly parallel, as we see in this figure.

The final step in finding the mean time to extinction is approximating the prefactor in Eq. (9). Following the approach in [49], we obtain
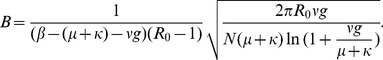
(12)


(We use Eq. (49) of [49] with 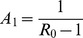, which is the value that corresponds to our case.) Note the dependence on the treatment parameters 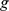 and 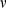.

**Figure 3:**
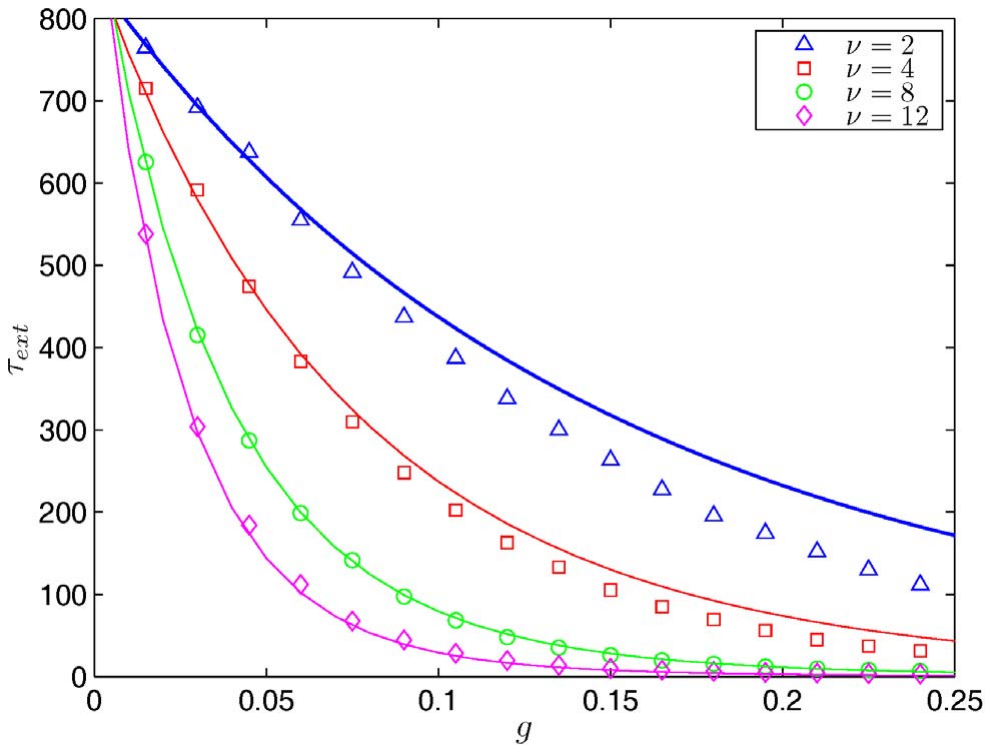
The effectiveness of various treatment combinations for Model 1. A plot of the mean time to disease extinction, 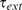 years, vs. the fraction of infected treated, 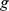, for different treatment frequencies, 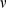 year^−1^. The results for the Monte Carlo simulations are averaged over 2,000 realizations and plotted as symbols. The curves of the same color show the approximation of the mean time to extinction by finding the action. The parameters are 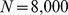 people and 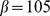 year^−1^. Note the exponential decrease in the mean time to extinction as the treatment fraction is increased.

To quantify the accuracy of the approximation to the mean time to extinction in Eq. (9), with 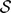 up to 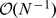, we compare it to the average extinction time found by a Monte Carlo simulation as described in Gillespie [50]. In Figure 3, the graph shows this comparison over a range of treatment percentages (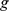) and frequencies (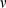). The simulation uses a population of 10,000 and we averaged the results of 2,000 realizations. As expected, the mean time to extinction decreases as the treatment percentage and frequency increase. Note the excellent agreement for small 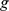, for which the asymptotic approximation was derived. Because the distribution of the mean extinction times is approximately exponential, the standard deviation of the data is equal to the mean. Therefore, as the mean decreases to zero, the standard deviation decreases and the prediction becomes more accurate. Also, we see an improvement in agreement as 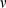 is increased. This is because the controlled rate to zero is faster with increasing 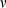, and the system has less time to relax back to the endemic state.

### Model 2

**Figure 4:**
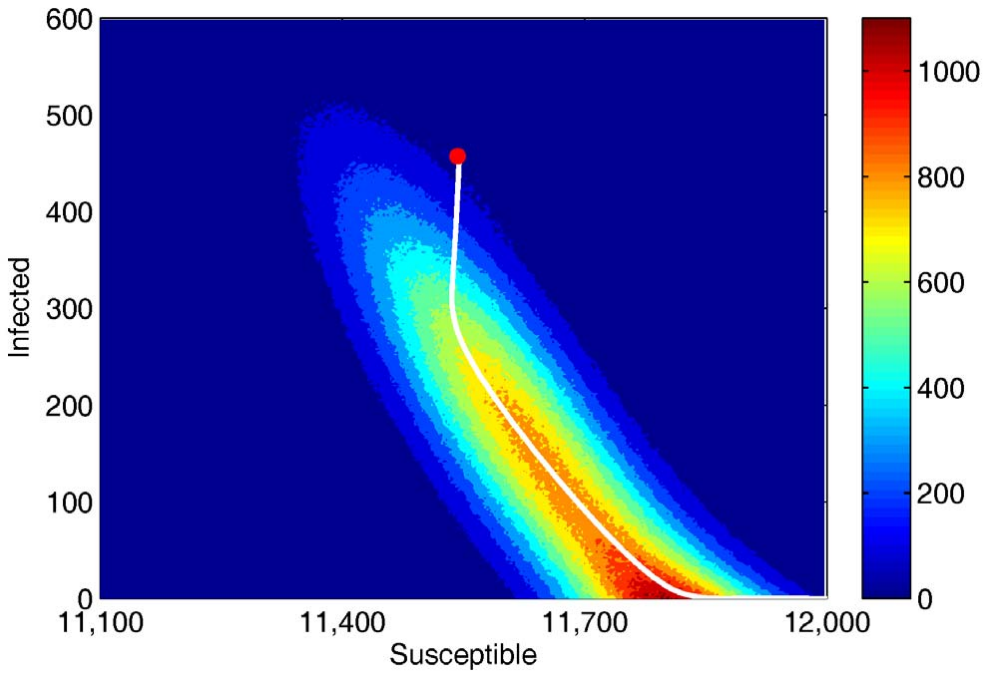
Probability density of extinction prehistory and the optimal path to extinction for Model 2. The red point denotes the endemic state. A simulation starts with the population at the endemic state and stops when the number of infected individuals is zero. The probability density uses the last five years of data from 200,000 Monte Carlo extinction realizations.The parameters are 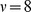 year^−1^, 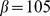 year^−1^, 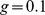, and 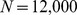 people. Red colors correspond to regions of highest frequency for the path to extinction. The optimal path (white curve) is computed from the Hamiltonian model and connects the endemic state to the extinct state. Notice that it lies on the peak of the probability density of extinction prehistory.

**Figure 5:**
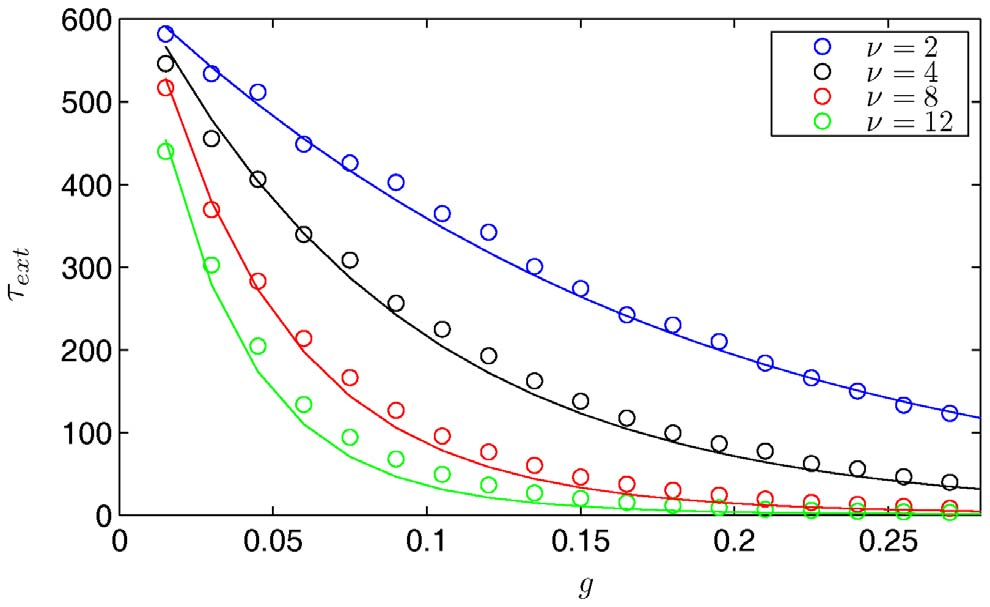
The effectiveness of various treatment combinations for Model 2. A plot of the mean time to extinction, 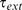, vs. the fraction of infected vaccinated during each treatment, 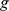, for different treatment frequencies, 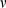 year^−1^. The averages of 2,000 Monte Carlo simulations are shown with symbols. The curves of the same color show the numerical approximation of 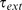 using the action 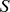 and a constant prefactor. For the parameters, we use 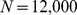 people and 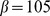 year^−1^.

The full SIS model has a Hamiltonian system in four dimensions and asymptotic approximations of the optimal path and action are not tractable. Therefore, we rely on numerical approximations. For example, we show the probability density of extinction prehistory and the optimal path to extinction in Figure 4. A simulation starts with the population at the endemic state and stops when the number of infected individuals is zero. The probability density uses the last five years of data from 200,000 Monte Carlo extinction realizations. Red colors correspond to regions of highest frequency for the path to extinction. The optimal path (white curve) is computed from the Hamiltonian model. Notice the agreement of the optimal path and the peak of the probability density of extinction prehistory. We also compare the approximation for the mean time to extinction given by the theory to data found by Monte Carlo simulation for small 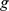 in Figure 5. There is an exponential decrease in the mean time to extinction as we increase the treatment, agreeing with the theory. Note that the standard deviation of the data is equal to the mean, as in Model 1, since the extinction times are exponentially distributed, approximately.

**Figure 6:**
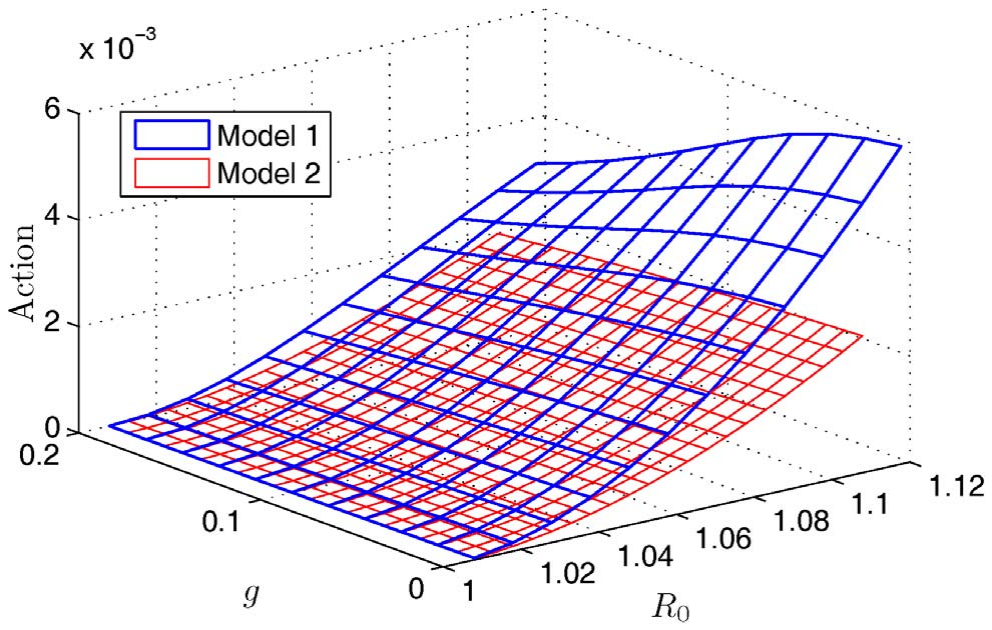
A comparison of the action approximations for Model 1 and Model 2. This plot shows the quantitative difference in the action approximation for Model 1 (blue) and Model 2 (red) as we vary 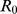 and 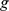. In this example, we use parameters 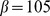 year^−1^, 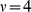 year^−1^, and 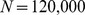.

We also comment on the differences in the constrained and unconstrained SIS models with treatment. In Figure 6, we compare the numerical approximations for actions of the two models. We see that they agree for small 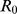, but the action for the constrained model increases much faster as both 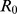 and 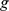 increase. This follows the result in [20], where the action for the constrained SIS model with no intervention was shown to have a logarithmic dependence on for 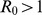. The constrained treatment model also follows the logarithmic scaling. In contrast, the unconstrained SIS model has an action which exhibits a quadratic power law dependence on 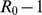. In addition, the theory can be used to avoid expensive simulations of long extinction times in large populations. The benefit of the full model is that it captures disease dynamics in a population with significant size fluctuations. The theory captures the rate of change in the mean time to extinction so that effectiveness in treatment schedules can be quantified.

## Discussion

In this paper, we quantified how treatment enhances the extinction of epidemics using a stochastic, discrete-population framework. Specifically, we based our study on a general formulation of an SIS model with treatment that is applied randomly in a Poisson fashion, accounting for the limited amount of resources. We used a WKB approximation to the master equation of the stochastic process to calculate the average time to extinction starting from the endemic state, as a function of the transmissibility of the disease and the strength and frequency of the treatment. We compared the extinction times obtained analytically and numerically from the WKB approximation with the values obtained from Monte Carlo simulations.

In addition, we explored the significance of the quasi-stationarity assumption that is fundamental to the WKB approximation. The existence of a quasi-stationary distribution peaked at the endemic point produces a meta-stable state in which the population fluctuates in a neighborhood around the same endemic point. In contrast, the extinct state lies in the exponentially small tail of the distribution. When a quasi-stationary distribution exists, the extinction of a disease is a rare event, i.e. the mean time to extinction is exponentially long. As we show in Text S1, the time to extinction is indeed exponentially long when the disease-free point lies in the tail of the distribution. The occurrence of extinction as a rare event means that the fluctuations exhibited by the random population dynamics are much smaller than an effective activation barrier. If the fluctuations are not small compared to the barrier, then the extinction events are not necessarily in the tail of the distribution, and hence not a rare event.

**Figure 7:**
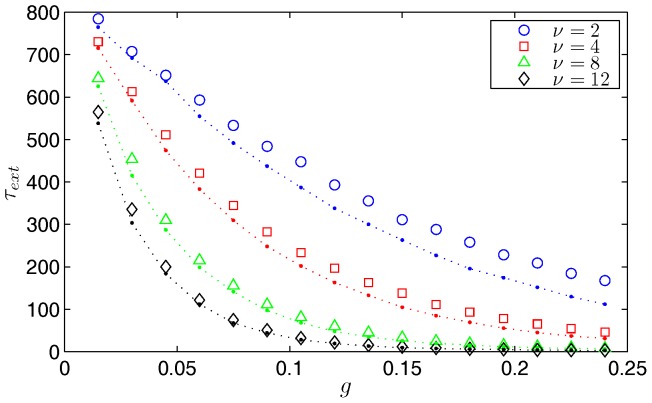
A comparison of periodic and random treatment effectiveness. For Model 1, a plot of the Monte Carlo simulated mean time to disease extinction for random (points connected by dotted lines) and periodic (symbols) treatment schedules vs. the fraction of infected vaccinated during each treatment. Results are shown for treatment frequencies, 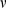 = 2, 4, 8, and 12 year^−1^ averaged over 2,000 realizations. The parameters are 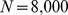 people and 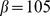 year^−1^. Note that the random treatment schedule has average extinction times consistently lower than the periodic treatment schedule.

Deterministic models of treatment are not accurate representations of the process in practice when applied to finite population realizations. A more realistic description is that, on average, treatment scheduling has a mean period or cycle, but is itself a random process. To quantify the difference between the deterministic and the stochastic descriptions, we compared the mean time to extinction for a strictly periodic and a Poisson-distributed treatment schedule obtained by averaging the Monte Carlo simulation results of many extinction events starting from the endemic state. We assume that a fraction 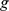 of the infected population is treated at a frequency of 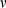 times per year and immediately return to a susceptible state. Therfore, the treatment is applied deterministically as a function of time and not simulated as a random event. In Figure 7, simulations support evidence that the random schedule had a faster mean time to extinction over the range of frequencies. The reason for this is that when the system is close to the extinct state, there is a benefit to having a number of treatment pulses in a short window of time; such a series of frequent treatment pulses are possible in the Poisson treatment scheduling but not in the deterministic one. Increasingly rapid pulses prevents the disease from relaxing to its endemic state, thereby enhancing the extinction rate.

**Figure 8:**
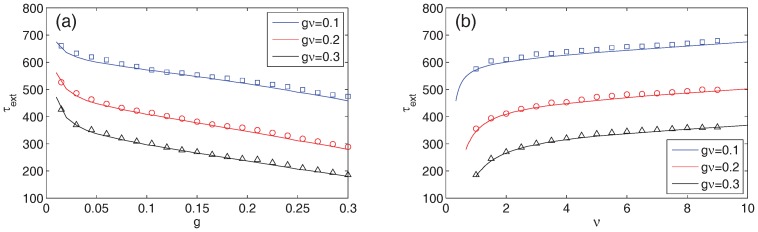
The effectiveness of various treatment combinations for a fixed treatment supply. Using Model 1 with a fixed treatment supply 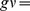
constant, we plot the mean time to extinction as a function of 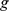 (panel a) or, alternatively, as a function of 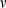 (panel b). The symbols represent the Monte Carlo simulation results for 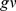 = 0.1, 0.2, and 0.3 averaged over 10,000 realizations. The curves represent the direct numerical solution of the associated master equation. The parameters are 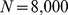 people and 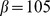 year^−1^. Note that the mean time to extinction is a decreasing function of 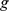 and an increasing one of 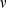.

The treatment program that we implement in our model has two degrees of freedom: the frequency 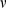 and the fraction of infected individuals that are treated, 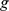. On average, there are 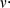
(1 year) treatment pulses each year and at each one, a number 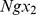 of infected individuals are treated, where 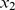 is the infected fraction at the moment each treatment pulse occurs. Supposing that there are a fixed number of treatment doses 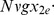
(1 year) = constant that may be applied each year (here 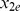 is the fraction of the population that is infected at the endemic point). A natural question that arises is the following: Given a fixed number of total treatment doses, how are 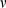 and 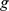 chosen so that the time to disease extinction is minimized. In both of our SIS models, the fixed number of treatment doses translates into 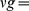 constant. Monte Carlo simulations of Model 1 show that, for given a fixed 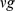 quantity, the mean time to extinction decreases as a function of 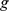, or alternatively, increases as a function of 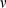 (Figure 8). The drop is particularly sharp for 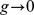. This appears to be a consequence of the rounding down of 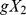 whenever a treatment pulse occurs (see Methods section). The treatment ceases to have an effect when there are less than 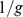 infecteds; for very small 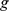, the threshold 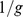 is significant when compared to the number of infecteds at the endemic state. Thus, the treatment helps to bring the number of infected down to 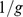, but not all the way to extinction. This issue does not appear if one instead chooses to round 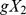 to the next-highest integer (results not shown). With this alternative method of rounding, the time to extinction actually has a sharp increase as 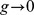. Monte Carlo simulations of Model 2 corroborate this finding. Thus, given a fixed number of resources, our stochastic simulations demonstrate that in order to eliminate infectious diseases, it is better to increase the pool of individuals reached by the treatment, rather than increase its frequency.

In conclusion, we have described a method to quantify the effectiveness of a random treatment program. We find that increasing the magnitude and frequency of randomly scheduled treatments provide an exponential decrease in average extinction times. We have presented evidence that supports how larger campaigns applied less frequently are the most effective in facilitating disease eradication. Several assumptions in the model clarify the accuracy of the analytic approximation to the mean time to extinction, but its exponential rate of decrease as we increase the intervention is consistent with simulations throughout our analysis as populations get very large. The techniques considered here can be easily generalized to other diseases, such as those that include seasonality or population structure. Future work in this area could provide a more targeted control strategy that would be robust in fluctuating environments as well as more efficient and economical disease eradication.

## Supporting Information

Figure S1
**Quasi-stationarity depicted through probability distributions.** Graphs of the WKB approximation of the SIS probability distributions using Eq. (3) for 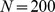. We show the case of 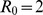, for which extinction is in the tail of the distribution. Conversely, extinction has a significant probability in the case of 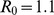. Note the height of the curve for 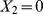. The dotted vertical lines show the location of the endemic state in 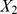 for each case.(TIFF)

Figure S2
**The drift of probability distributions for systems without quasi-stationarity.** A plot of the solution of the one-dimensional master equation in with 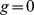 over time using the distribution from the WKB approximation, Eq. (3), as the initial condition. For 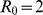 (panel a), the extinct state lies in the tail of the distribution and a quasi-stationary distribution exists. Extinction occurs only over exponentially long times. For 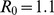 (panel b) the endemic state is close to the absorbing boundary and extinction is not a rare event. The absorption of this distribution into the boundary is apparent.(TIFF)

Text S1 **Supporting Information: Quasi-stationarity.**
(PDF)
