# Supplementary figures and images for: Intervention-Based Stochastic Disease Eradication
Source: PLoS One. 2013 Aug 5;8(8):e70211. doi: 10.1371/journal.pone.0070211 (PMC3734278; doi:10.1371/journal.pone.0070211)

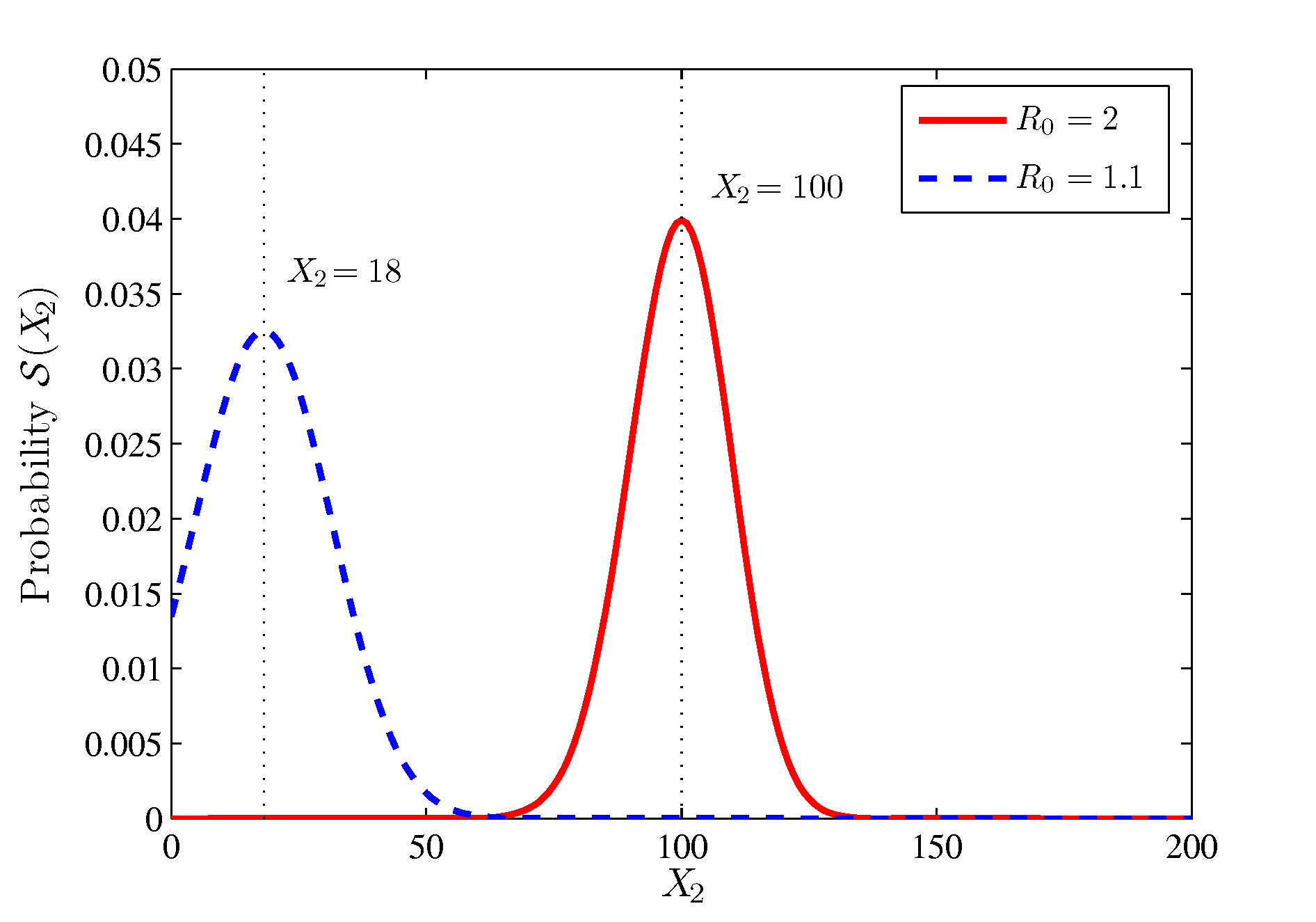

Supplement: Figure S1 — Quasi-stationarity depicted through probability distributions. Graphs of the WKB approximation of the SIS probability distributions using Eq. (3) for . We show the case of , for which extinction is in the tail of the distribution. Conversely, extinction has a significant probability in the case of . Note the height of the curve for . The dotted vertical lines show the location of the endemic state in for each case. (TIFF) [file pone.0070211.s001.tiff]

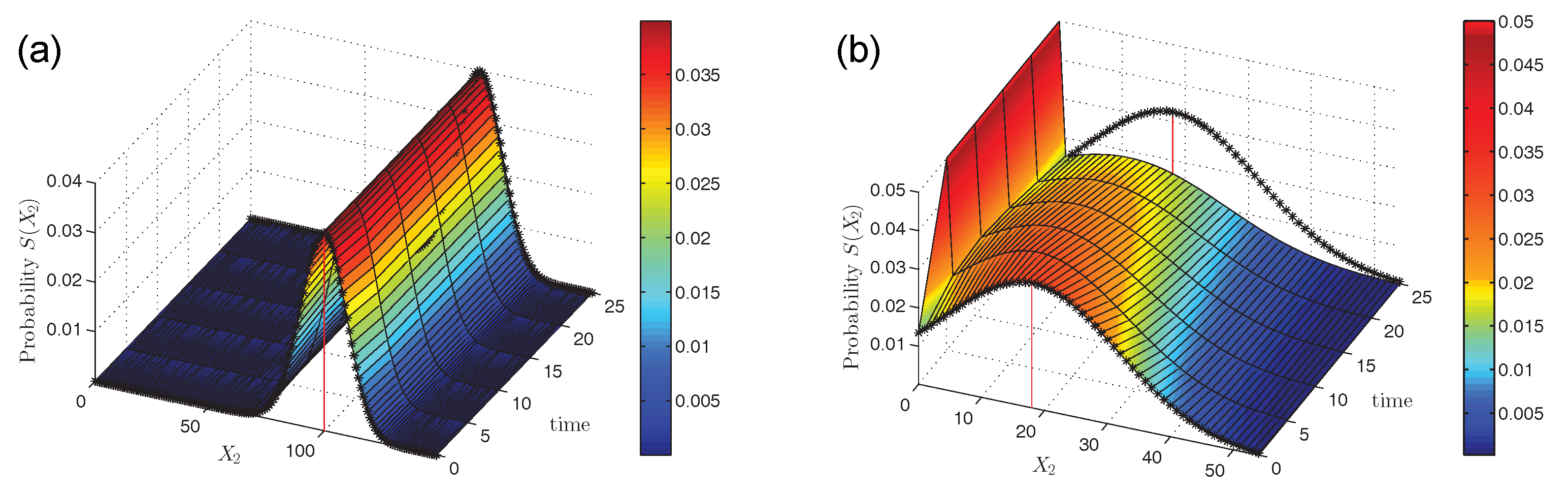

Supplement: Figure S2 — The drift of probability distributions for systems without quasi-stationarity. A plot of the solution of the one-dimensional master equation in with over time using the distribution from the WKB approximation, Eq. (3), as the initial condition. For (panel a), the extinct state lies in the tail of the distribution and a quasi-stationary distribution exists. Extinction occurs only over exponentially long times. For (panel b) the endemic state is close to the absorbing boundary and extinction is not a rare event. The absorption of this distribution into the boundary is apparent. (TIFF) [file pone.0070211.s002.tiff]
