# Supplementary material for: Intervention-Based Stochastic Disease Eradication
Source: PLoS One. 2013 Aug 5;8(8):e70211. doi: 10.1371/journal.pone.0070211 (PMC3734278; doi:10.1371/journal.pone.0070211)
Supplement: Text S1 — Supporting Information: Quasi-stationarity. (PDF) [file pone.0070211.s003.pdf]

Supporting Information: Quasi-stationarity

In this section, we illustrate the central importance of the quasi-stationarity assumption for the accuracy of the WKB expression for the mean-extinction time. For this, we numerically compute solutions to the master equation for the constrained SIS model without treatment (Model 1 with $g = 0$), and determine when the WKB condition $N\mathcal{S}_{opt} \gg 1$ holds and when it does not by comparing the outcome with the WKB results.

In the normalized constrained variables where $x_1+x_2 = 1$ (i.e., no fluctuations in the total population), the Hamiltonian takes the form:

$$H(x_2, p_2) = \beta x_2(1 - x_2)(e^{p_2} - 1) + (\mu + \kappa)x_2(e^{-p_2} - 1). \tag{1}$$

The Hamiltonian system under the constraint $H(x_2, p_2) = 0$ has the steady states

$$(x_{2e}, p_{2e}) = \left(1 - \frac{1}{R_0} - \frac{\nu g}{\beta}, 0\right) \tag{2}$$

and $(x_{20}, p_{20}) = (0, -\ln R_0)$, which denote the endemic and extinct states, respectively. The system also possesses a third steady state $(x_{2m}, p_{2m}) = (0, 0)$. The attracting states (x_{2e}, p_{2e}) and (x_{2m}, p_{2m}) correspond to the zero fluctuation states that exist in the mean field equation (deterministic system). Since there is a nonzero probability current at the extinct state, (x_{20}, p_{20}) is a new state created by the noise in the system. We also note that for finite populations noise is not generally known due to the random interactions of individuals. However, in this model, the noise-free extinct state is accessible if the noise is known to be Gaussian.

The most probable path from the endemic to the extinct point is the heteroclinic trajectory connecting the fixed point (x_{2e}, p_{2e}) with the fluctuational extinction point (x_{20}, p_{20}). The optimal path is given by $p_2(x_2) = -\ln(R_0(1 - x_2))$. Therefore, the action from the endemic state to a point x_2 along the optimal path up to the zeroth order of N is

$$\mathcal{S}(x_2) = \int_{\frac{R_0-1}{R_0}}^{x_2} -\ln(R_0(1 - x_2'))\, dx_2'. \tag{3}$$

In particular, the action from the endemic state to the extinct state along the optimal path is

$$\mathcal{S}_{opt} = \mathcal{S}(0) = -1 + \ln(R_0) + \frac{1}{R_0}. \tag{4}$$

Since $R_0 > 1$, for sufficiently large N we have $N\mathcal{S}_{opt} \gg 1$, ensuring the extinct point lies in the tail of the probability distribution, where its value $\rho(0) = Ae^{-N\mathcal{S}_{opt}}$ is exponentially small.

In Figure S1, the WKB approximations to the quasi-stationary distribution are shown for the cases of $R_0 = 2$ and $R_0 = 1.1$. The value of the distribution at zero shows that the extinction point is definitely not in the tail of the distribution for $R_0 = 1.1$ and hence does not constitute a rare event. In contrast, for $R_0 = 2$, the extinct state is in the tail of the distribution and hence we expect the WKB result to be accurate.

Because the disease free equilibrium is always an absorbing boundary in the one-dimensional case, the system decays to the extinct state in the long term. If a quasi-stationary distribution exists, the complete decay happens for exponentially long times; otherwise, it occurs on a much shorter time-scale. To illustrate this phenomenon, we show the numerical solution of the master equation over time in Figure S2. The initial probability distribution at $t = 0$ is set to the WKB approximation of the SIS probability distributions using Eq. (3). The absorption into the disease-free state is apparent in the $R_0 = 1.1$ case (panel b), but completely imperceptible for $R_0 = 2$ (panel a) over the time-scale shown.
